# Sex Under the Influence of Drugs Among People Who Use Drugs in Cambodia: Findings From a National Survey

**DOI:** 10.1007/s10508-021-02243-x

**Published:** 2022-02-22

**Authors:** Chan Hang Saing, Pheak Chhoun, Navy Chann, Ponha Uk, Phalkun Mun, Sovannary Tuot, Siyan Yi

**Affiliations:** 1 Saw Swee Hock School of Public Health, National University of Singapore and National University Health System, 12 Science Drive 2, #10-01, Singapore, 117549 Singapore; 2 KHANA Center for Population Health Research, Phnom Penh, Cambodia; 3 National Center for HIV/AIDS, Dermatology and STD, Phnom Penh, Cambodia; 4 Center for Global Health Research, Touro University California, Vallejo, CA USA

**Keywords:** Sex and drugs, Sexual behavior, HIV, Resource-limited setting, Asia, Substance use disorders

## Abstract

Studies in drug use settings rarely use sex under the influence of drugs as an indicator of sexual risk behaviors. This study explored the prevalence of sex under the influence of drugs and its correlates among people who use drugs (PWUD) in Cambodia. We included 1147 PWUD from 12 provinces in this study. A multiple logistic regression analysis was conducted to identify factors associated with sex under the influence of drugs. Of the total, 39.7% reported having had sex under the influence of drugs in the past three months. After adjustment, sex under the influence of drugs was significantly associated with living in urban areas (AOR 2.97, 95% CI 1.68–5.27), having two to three (AOR 2.48, 95% CI 1.76–3.49) and four or more sexual partners (AOR 6.46, 95% CI 4.24–9.85), engaging in transactional sex (AOR 1.69, 95% CI 1.19–2.39), using methamphetamine (AOR 2.97, 95% CI 2.06–4.31), using drugs for three years or longer (AOR 1.67, 95% CI 1.15–2.41), having been to a drug rehabilitation center (AOR 1.77, 95% CI 1.18–2.41), having a network of ten or more PWUD (AOR 1.82, 95% CI 1.25–2.66), and having high psychological distress (AOR 1.66, 95% CI 1.25–2.22). This study documents the high prevalence of sex under the influence of drugs and its risk factors among male and female PWUD in Cambodia. These findings point to the need for integrating HIV and harm-reduction programs using innovative approaches to address the overlapping risks in this key population.

**Supplementary Information:**

The online version contains supplementary material available at 10.1007/s10508-021-02243-x.

## Introduction

People who use drugs (PWUD), including people who inject drugs, are highly exposed to sexual risk behaviors, such as sex under the influence of drugs. Sex under the influence of drugs is defined as sexual intercourse when a partner is intoxicated with one of the drugs, including heroin, cannabis, amphetamines, cocaine, and benzodiazepines (Ding et al., 2013; Guimarães et al., 2016; Ross et al., 1994; Zhao et al., 2019). This behavior puts PWUD at a higher risk of the human immunodeficiency virus (HIV) and other sexually transmitted infections than the general population (Armstrong et al., 2013; Mackesy-Amiti et al., 2014). Therefore, prevention and reduction of sex under the influence of drugs have the potential to ameliorate the HIV epidemic and improve the health of this key population.

Globally, studies on the association between drug use and sexual risk behaviors among HIV key populations tend to focus more on transactional and condomless sex than sex under the influence of drugs. For instance, a street-intercept survey of 888 adults in two inner-city communities with a high HIV prevalence in the United States showed that people who used crack cocaine were more likely to trade sex for money or drugs than people who did not use drugs (Baseman et al., 1999). A study among young African American men who have sex with men documented a positive association between drug use before sexual activity and inconsistent condom use or multiple sexual partnerships (Browne et al., 2009). In the same manner, another study showed that the increase in severity of drug use had a positive association with the increased frequency of unprotected sex among American men in substance use treatment (Newville et al., 2018). Similar evidence has also been observed among PWUD in Brazil (Guimarães et al., 2016), Cambodia (Coupland et al., 2019; Mburu et al., 2019; Page et al., 2019), China (Ding et al., 2013; Zhao et al., 2019), India (Suohu et al., 2012), and the Netherlands (Spauwen et al., 2015). A recent thematic review showed that drug use facilitated people’s willingness to engage in different types of sex, including undesired and risky ones (Smith et al., 2020).

A few other studies looked at sex under the influence of drugs as a risk factor of sexual risk behaviors. For instance, studies in different settings have reported the association between sex under the influence of drugs and the increased engagement in sexual risk behaviors, such as condomless sex, transactional sex, and group sex (Guimarães et al., 2016; Leigh & Stall, 1993; Leigh et al., 2008; Newville et al., 2018; Smith et al., 2020; Zule et al., 2007). The authors explained that, under the influence of drugs, a partner would lose judgment and proper decision-making on or negotiation for safe sex.

The high prevalence of sex under the influence of drugs among PWUD deserves further research given its strong association with sexual risk behaviors and the limited number of studies on this topic. A study in Sydney, Australia, found that sex under the influence of drugs among people who inject drugs was associated with having a lower likelihood of being in drug treatment, having a higher number of sexual partners, sharing injection equipment with other people, being intoxicated when injecting, and being not sex workers (Ross et al., 1994). In a study in the United States, 73% of men who use drugs experienced sex under the influence of drugs in the past three months, and 39% adopted sex under the influence of drugs in their most recent sexual intercourse (Calsyn, Cousins, et al., 2010; Calsyn, Crits-Christoph, et al., 2010). Another study among 901 PWUD in two detoxication centers in Jiangsu province in China found that around 47% of the participants engaged in sex under the influence of drugs either sometimes or often over the past year before being admitted to the centers (Zhao et al., 2019).

The evidence of the association between drug use and sexual risk behaviors in HIV key populations in Cambodia has been well documented (Coupland et al., 2019; Mburu et al., 2019; Page et al., 2019). However, no studies have examined risk factors associated with sex under the influence of drugs among PWUD. The HIV prevalence among people who use injecting and non-injecting drugs in 2017 was high at 15% and 6%, respectively (Tuot et al., 2019; Yi et al., 2019, 2020). A recent study examined sexual risk behaviors among male and female adults who use drugs in Cambodia, but sex under the influence of drugs was not an outcome measure (Ngor et al., 2019). The study showed that about 60% of male participants were involved in transactional sex, and 46% reported inconsistent condom use. Therefore, this study aims to fill this gap and contribute to the international literature by exploring the prevalence and associated factors of sex under the influence of drugs among PWUD in Cambodia. Our results will inform future policy of the prevention of sexual risk behaviors in this HIV key population.

## Method

### Participants

This cross-sectional study used a subsample of 1,147 participants, who reported whether they had sex under the influence of drugs in the past three months, from the National Integrated Biological and Behavioral Survey (IBBS) of 1,677 PWUD in Cambodia. We excluded participants who reported that they did not have sexual intercourse and did not use any illicit drugs in the past three months. TThe primary objective of the IBBS survey was to estimate the HIV prevalence among PWUD in Cambodia and examine knowledge, attitude, and practices of illicit drug use behaviors and sexual risk behaviors (Tuot et al., 2019; Yi et al., 2019, 2020). The survey was conducted between June and December 2017 in 12 provinces, where 21 operational districts with a high HIV and drug use burden were located. In the IBBS, PWUD were defined based on the Cambodian Law on Control of Drugs. They referred to individuals who used any illegal injecting or non-injecting drugs in the last 12 months. Individuals eligible for the survey must be at least 18-years-old, meet the survey definition of PWUD, be able to communicate in Khmer, and voluntarily provide written informed consent to participate in the study.

### Procedure

Given the PWUD population’s hidden nature, a peer-based social network recruitment method was used to recruit the participants in the 21 study locations–six of which were in the capital city of Phnom Penh. The recruitment procedures followed four steps. First, four eligible seeds with an established social network of at least three PWUD in each study location were identified with assistance from non-governmental organizations working in the data collection sites. Second, a personal identification number was then assigned to each participant who had signed the consent form. Third, each seed was provided with three coupons for referring three other PWUD in their network to the study. The seed received US$2 for each successful referral and was expected to recruit between three and six participants. Finally, the recruited participants were offered an opportunity to be a seed and refer other peers to the study using coupons provided by the study team.

Two data collection teams were formed. Each team comprised one field supervisor, five interviewers, one lab technician, and one counselor. The teams were trained about data collection procedures, informed consent process, interview techniques, participants' confidentiality and privacy protection, and questionnaire administration practices. The teams were also required to review the study protocol and data collection tools. The team leaders also reviewed their team members' performance daily and addressed issues during the fieldwork. Before the interview, the counselor explained the potential risk and benefits of the study to the participants and obtained written informed consent from the participants. Each interviewed participant received US$5 for their time and transportation compensation.

### Measures

We adapted existing tools from our previous studies on HIV key populations in Cambodia to develop this survey questionnaire (Sopheab et al., 2014; Yi et al., 2014, 2016). A workshop for the questionnaire validation was conducted with participation from representatives of communities and stakeholders working on HIV and harm reduction. The questionnaire was pretested to ensure that the contents were suited to the context, and the data collection teams were adequately able to smoothly conduct the data collection.

We designed the questionnaire to collect sociodemographic information, including residence types (rural or urban), sex, age, ethnicity, marital status, education, living arrangement, occupation, and income. The questionnaire also collected information on sexual behaviors, such as the number of sexual partners, frequency of condom use, having sex in exchange for money or goods, and having sex under the influence of drugs with any partner in the past three months. We categorized the number of sexual partners into three groups based on its distribution. To define “sex under the influence of drugs,” we asked a single yes/no question, “In the past three months, have you ever had sexual intercourse when you or your partners were intoxicated with drugs?”

The survey also collected information related to substance use behaviors, such as the type of drugs used, duration of drug use, experience in a drug rehabilitation center, and the size of the network of drug users they knew. We dichotomized methamphetamine and heroin use separately and grouped the duration of drug use based on its distribution. Similarly, we grouped the number of drug users that the participants knew into three groups based on its distribution. We used the 12-item General Health Questionnaire (GHQ-12) to measure psychological distress (Goldberg, 1972). The four-point Likert-like scale was re-coded using a “0–0-1–1” method to generate a dichotomous variable. Individuals who had the sum score above the mean (cut-off) were considered to have high psychological distress.

### Data Analyses

We used Stata (Stata Corp LP, version 14.1) for the statistical analyses. We built a multiple logistic regression model to identify factors associated with sex under the influence of drugs, following several steps. First, we grouped continuous variables based on their respective distributions. Next, bivariate analyses were conducted using the chi-square test to examine the association between sex under the influence of drugs and variables selected based on previous studies. Variables independently associated with sex under the influence of drugs at *p*-values < 0.25 were simultaneously included in a multiple logistic regression model. The model included drug use duration instead of age because the two independent variables affected the outcome similarly and were correlated. We presented odds ratios (OR) of the outcome for each covariate to show the direction of and the change in the association’s magnitude. Finally, we presented adjusted odds ratios (AOR) in the multiple logistic regression model with their respective 95% confidence intervals (CI). We also performed sensitivity analyses to see whether results for men differed from those for women, as shown in the literature (Calsyn et al., 2010a, b; Scott-Sheldon et al., 2009; Zhao et al., 2019).

Ethical approval was obtained from the National Ethics Committee for Health Research (NECHR) of the Ministry of Health in Cambodia (No. 420 NECHR). We removed all personal identifiers from the data collection documents. Participants were informed of the risks and benefits of their participation and the study’s voluntary nature. The study participants provided written informed consent before the data collection started.

## Results

### Sociodemographic Characteristics

Table 1 shows the sociodemographic characteristics of the study participants. The participants’ mean age was 28.6 years (SD 7.9), with an average monthly income in the past six months of US$113.2 (SD 75.3). Of the total participants, 39.7% (39.6% for men vs. 40.0% for women) reported sex under the influence of drugs in the past three months. The majority (87.9%) of the participants lived in urban areas, and slightly less than half were women. More than two-thirds (78.1%) were younger than 35-years-old, and 43.9% were never married. More than half (54.6%) had attained no or only primary education. One in ten participants (9.8%) lived on the street (homeless), and 9.8% were unemployed. Almost one in five (18.4%) were entertainment workers. Around one-third (33.2%) earned an average monthly income of less than US$100 in the past three months.

**Table 1:** Sociodemographic characteristics of people who use drugs with and without sex under the influence of drugs in the past 3 months

Sociodemographics	Sex under the influence of drugs			
	Total *(n* = 1147)	Yes (*n* = 456)	No (*n* = 691)	
	*n* (%)	*n* (%)	*n* (%)	*p*-value^*^
Living in urban area	1008 (87.9)	436 (95.6)	572 (82.8)	< 0.001
Female	472 (41.1)	189 (41.4)	283 (40.9)	0.86
Age groups				0.04
18–24	382 (33.3)	132 (29.0)	250 (36.2)	
25–34	513 (44.8)	216 (47.5)	297 (43.0)	
35 +	251 (21.9)	107 (23.5)	144 (20.8)	
Khmer ethnicity	1087 (95.3)	428 (94.7)	659 (95.6)	0.45
Marital status				0.26
Married	455 (39.9)	174 (38.4)	281 (41.0)	
Divorced/separated/widowed	184 (16.2)	83 (18.3)	101 (14.7)	
Never married	499 (43.9)	196 (43.3)	303 (44.2)	
Level of education				0.48
Primary (grades 0–6)	626 (54.6)	256 (56.1)	370 (53.5)	
Lower secondary (grades 7–9)	311 (27.1)	124 (27.2)	187 (27.1)	
Upper secondary or higher	210 (18.2)	76 (16.7)	134 (19.4)	
Living arrangement				0.07
With family	491 (42.9)	193 (42.4)	298 (43.2)	
Homeless	112 (9.8)	58 (12.7)	54 (7.8)	
Own home	273 (23.8)	99 (21.7)	174 (25.2)	
With friends	136 (11.9)	56 (12.3)	80 (11.6)	
Others	132 (11.6)	49 (10.9)	83 (12.2)	
Employment				0.14
Unemployed	112 (9.8)	55 (12.1)	57 (8.3)	
Entertainment worker	211 (18.4)	76 (16.7)	135 (19.5)	
Office/laborer/farmer	428 (37.3)	167 (36.6)	261 (37.8)	
Others	396 (34.5)	158 (34.6)	238 (34.4)	
Level of monthly income in USD				0.78
< 100	381 (33.2)	156 (34.2)	225 (32.5)	
100–199	550 (48.0)	213 (46.7)	337 (48.8)	
200 +	216 (18.8)	87 (19.1)	129 (18.7)	

^*^Chi-square (or Fisher’s exact test when a cell count was smaller than 5) was used

### Characteristics of Sexual Behaviors

Table 2 shows that 36.6% of the participants had their first sex when they were younger than 18. Half reported having sex with two or more partners (49.9%), and only 24.9% reported consistently using condoms in the past three months. About one-third (35.8%) reported having sex in exchange for money or gifts in the past three months. A significantly higher proportion of the participants who reported having sex under the influence of drugs had their first sex before they turned 18-years-old (42.7% vs. 32.5%, *p* < 0.001), had sex with at least four partners (37.4% vs. 12.0%, *p* < 0.001), and had sex in exchange for money or goods (49.3% vs. 26.9%, *p* < 0.001) in the past three months.

**Table 2:** Sexual behaviors, substance use, and psychological distress among people who use drugs with and without sex under the influence of drugs in the past 3 months

	Sex under the influence of drugs			
	Total *(n* = 1147)	Yes (*n* = 456)	No (*n* = 691)	
	*n* (%)	*n* (%)	*n* (%)	*p*-value
Number of sex partners in the past 3 months (*n* = 1134)	< 0.001			
One partner	568 (50.1)	137 (30.3)	431 (63.2)	< 0.001
2–3 partners	315 (27.8)	146 (32.3)	169 (24.8)	< 0.001
4 + partners	251 (22.1)	169 (37.4)	82 (12.0)	< 0.001
Age at first sex < 18 (*n* = 1139)	417 (36.6)	194 (42.7)	223 (32.5)	< 0.001
Always used condoms in the past 3 months (*n* = 1146)	285 (24.9)	114 (25.0)	171 (24.8)	0.93
Having sex in exchange for money or gift in the past 3 months (*n* = 1147)	411 (35.8)	225 (49.3)	186 (26.9)	< 0.001
Type of drug used in the past three months (*n* = 1147)				
Methamphetamine	850 (74.1)	397 (87.1)	453 (65.5)	< 0.001
Heroin	113 (9.8)	54 (11.8)	59 (8.5)	0.07
Duration of drug use (*n* = 1147)				< 0.001
Less than one year	382 (33.3)	117 (25.7)	265 (38.4)	< 0.001
1–2 years	314 (27.4)	112 (24.6)	202 (29.2)	0.08
3 years or more	450 (39.3)	226 (49.7)	224 (32.4)	< 0.001
Been sent to drug rehabilitation in a life time (*n* = 1147)	183 (15.9)	112 (24.5)	71 (10.3)	< 0.001
Number drug users you know in the past 12 months (*n* = 1147)	< 0.001			
0–4 people	315 (27.5)	81 (17.7)	234 (33.8)	< 0.001
5–9 people	318 (27.7)	106 (23.2)	212 (30.7)	< 0.001
10 + people	514 (44.8)	269 (59.1)	245 (35.5)	< 0.001
High level of psychological distress^†^ (*n* = 1147)	509 (44.4)	252 (55.3)	257 (37.2)	< 0.001

*GHQ* General Health Questionnaire, *PWUD* people who use drugs

^*^Chi-square test (or Fisher’s exact test when a cell count was smaller than 5) was used

^†^Participants with the sum score of GHQ-12 above its mean were considered to have high psychological distress

### Substance Use and Psychological Distress

As shown in Table 2, 74.1% of the study participants reported using methamphetamine and 9.8% using heroin in the past three months. One-third (33.3%) reported using drugs for less than one year, while 39.3% using them for three years or more. Less than one in five (15.9%) reported having been to a rehabilitation center. Slightly less than half (44.8%) reported having a network of ten or more PWUD, and 44.4% had high psychological distress. A significantly higher proportion of the participants who reported having sex under the influence of drugs in the past three months used methamphetamine (87.1% vs. 65.5%, *p* < 0.001), used drugs for at least three years (49.7% vs. 32.4%, *p* < 0.001), had been to a drug rehabilitation center (24.5% vs. 10.3%, *p* < 0.001), had a network of ten or more PWUD (59.1% vs. 35.5%, *p* < 0.001), and had high psychological distress (55.3% vs. 37.2%, *p* < 0.001).

### Factors Associated with Sex Under the Influence of Drugs

Table 3 shows the results of the bivariate and multiple logistic regression analyses. The odds of having sex under the influence of drugs were significantly higher in participants living in urban areas (AOR 2.97, 95% CI 1.68–5.27) relative to those living in rural areas but significantly lower in participants who were entertainment workers (AOR 0.36, 95% CI 0.19–0.65) relative those who were unemployed. The odds were also significantly higher in participants having two to three (AOR 2.48, 95% CI 1.76–3.49) and four or more (AOR 6.46, 95% CI 4.24–9.85) sexual partners relative to those having only one partner in the past three months.

**Table 3:** Risk factors associated with sex under the influence of drugs in the past 3 months among people who use drugs in bivariate and multiple logistic regression analyses

Variables in the model	OR (95% CI)	*P*-value	AOR (95% CI)	*p*-value^*^	
*Living area*					
Rural	Reference		Reference		
Urban	4.53 (2.78–7.40)	< 0.001	2.97 (1.68–5.27)	< 0.001	
*Living arrangement*					
With family	Reference		Reference		
Homeless	1.66 (1.09–2.50)	0.02	1.41 (0.84–2.37)	0.19	
Own home	0.87 (0.65–1.19)	0.41	1.15 (0.79–1.68)	0.45	
With friends	1.08 (0.73–1.59)	0.69	1.18 (0.73–1.91)	0.50	
Others	0.91 (0.61–1.35)	0.65	0.72 (0.45–1.15)	0.17	
*Employment*					
Unemployed	Reference		Reference		
Entertainment worker	0.58 (0.37–0.93)	0.02	0.36 (0.19–0.65)	0.001	
Office/laborer/farmer	0.66 (0.44–1.00)	0.41	0.64 (0.39–1.05)	0.08	
Others	0.69 (0.45–1.04)	0.08	0.48 (0.29–0.79)	0.004	
*Number of sex partners in past three months*					
One partner	Reference		Reference		
2–3 partners	2.71 (2.03–3.64)	< 0.001	2.48 (1.76–3.49)	< 0.001	
4 + partners	6.48 (4.68–8.98)	< 0.001	6.46 (4.24–9.85)	< 0.001	
*Age at first sex*					
Below 18-years-old	Reference			Reference	
18 years and above	0.65 (0.51–0.83)	< 0.001	0.87 (0.65–1.18)	0.38	
*Having sex in exchange for money or gift in past 3 months*					
No	Reference		Reference		
Yes	2.64 (2.06–3.39)	< 0.001	1.69 (1.19–2.39)	0.003	
*Methamphetamine use in the past three months*					
No	Reference		Reference		
Yes	3.53 (2.58–4.84)	< 0.001	2.97 (2.06–4.31)	< 0.001	
*Heroin use in the past three months*					
No	Reference		Reference		
Yes	1.44 (0.97–2.12)	0.07	0.97 (0.58–1.64)	0.92	
*Duration of drug use*					
Less than one year	Reference		Reference		
1–2 years	1.25 (0.91–1.72)	0.16	1.34 (0.91–1.95)	0.13	
3 years or more	2.28 (1.72–3.04)	< 0.001	1.67 (1.15–2.41)	0.007	
*Been sent to drug rehabilitation in a lifetime*					
No	Reference		Reference		
Yes	2.84 (2.05–3.93)	< 0.001	1.77 (1.18–2.67)	0.006	
*Number drug users you know in the past 12 months*					
0–4 people	Reference		Reference		
5–9 people	1.44 (1.02–2.04)	0.04	1.22 (0.81–1.83)	0.34	
10 + people	3.17 (2.33–4.31)	< 0.001	1.82 (1.25–2.66)	< 0.002	
*Level of psychological distress*^*†*^					
Low	Reference		Reference		
High	2.08 (1.64–2.65)	< 0.001	1.66 (1.25–2.22)	0.001	

*CI* confidence interval, *AOR* adjusted odds ratio, *OR* odds ratio

^*^Variables associated with sex under the influence of drugs in the bivariate analyses at a level of p < 0.05 were simultaneously included in the model

^†^Participants with the sum score of the General Health Questionnaire (GHQ-12) above its mean were considered to have high psychological distress

The odds of having sex under the influence of drugs were significantly higher in participants who reported having sex in exchange for money or goods (AOR 1.69, 95% CI 1.19–2.39) and participants who used methamphetamine (AOR 2.97, 95% CI 2.06–4.31) in the past three months relative to those who did not. Similarly, the odds of having sex under the influence of drugs were significantly higher among participants who had used drugs for more than three years relative to those who used drugs for less than one year (AOR 1.67, 95% CI 1.15–2.41), and participants who had been to a drug rehabilitation center relative to those who had not (AOR 1.77, 95% CI 1.18–2.67). Last, the odds of having sex under the influence of drugs were significantly higher in participants with a network of at least ten PWUD than those with a network of four or less PWUD (AOR 1.99, 95% CI 1.38–2.88), and participants who had high psychological distress relative to those with low psychological distress (AOR 1.66, 95% CI 1.25–2.22).

### Sensitivity Analyses

We reran Tables 1–3 for male and female subgroups separately to examine whether these results differed from those in the overall sample (See Tables S1, S2, S3, S4, S5, and S6 in the Supplementary Materials). Results in multivariable analyses were quite similar in men, but the results were moderately different in women from those in the overall sample.

## Discussion

This study documented a high prevalence of sex under the influence of drugs among PWUD in Cambodia at 39.7%. The prevalence was lower than the 46.6% prevalence found in China (Zhao et al., 2019), approximately 50% in Australia (Ross et al., 1994), and 76% in the United States (Calsyn et al., 2010a, b). We did not find the differences in the prevalence of sex under the influence of drugs in males and females, indicating that female PWUD broke the gender norms that limited their freedom from engaging in drugs and sex (Fordham, 2003; Mai & Kittisuksathit, 2019; UNFPA, 2016). This finding is unique to Cambodia since it departed from findings in the literature (Scott-Sheldon et al., 2009; Zhao et al., 2019).

PWUD living in urban areas exhibited a higher probability of having sex under the influence of drugs than their rural peers. Urbanization could have facilitated this association. The share of the urban population in Cambodia's total population increased from 19% in 2007 to 23.0% in 2017 (Ritchie & Roser, 2018). Evidence of the linkage between urbanization and increased drug availability and use has been documented (Paykel et al., 2000; Sundquist & Frank, 2004). Moreover, in this study, larger proportions of urban PWUD consumed either methamphetamines or heroin than that of rural PWUD. Urban PWUD also had a relatively larger number of sexual partners and a PWUD network. Our findings point to the need for an increase in community-based harm-reduction services for PWUD in urban areas.

We found that sex under the influence of drugs was associated with the number of sex partners, consistent with the findings in previous studies. For instance, a study among people who inject drugs in Australia identified the number of sexual partners as a predictor of sex under the influence of drugs (Ross et al., 1994). Similar results were found in men and women. This finding could be attributed to the number of PWUD whom the participants had known. In this study, the participants with a PWUD network of 10 or more were twice as likely to engage in sex under the influence of drugs. These results suggest that future interventions aiming to prevent sex under the influence of drugs, and HIV infection should devise a strategy to identify and target PWUD with multiple sexual partners and an extensive PWUD network.

Sex in exchange for money or goods was associated with sex under the influence of drugs, consistent with findings in a study in Iran (Kamel-Khodabandeh et al., 2018), but not with findings in a study in Australia (Ross et al., 1994). Our subgroup analyses confirmed the association in men but not in women. This finding was surprising since close to half of women engaged in the entertainment sector in which transactional sex was more common (Brody et al., 2019). We speculated that long-term drug use might have mediated the association among male participants as close to half of the men who had transactional sex had been using drugs for at least three years. Our finding highlights the need for interventions that target male long-term drug users to reduce the prevalence of sex under the influence and prevent HIV infections.

Furthermore, methamphetamine use was associated with sex under the influence of drugs. Similar results were found in both men and women. The relationship between drug use, methamphetamine use in particular, and sexual risks in HIV key populations in Cambodia have also been reported (Coupland et al., 2019; Mburu et al., 2019; Page et al., 2019). However, other types of drugs are also associated with sexual risk behaviors in other settings. For instance, McKeganey and Banard (1992) noted that some drugs such as temazepam made individuals too intoxicated to take sexual precautions, while Wachter et al. (1992) and Rawson et al. (2002) reported the association between sexual risks and crack cocaine use. These findings suggest that the association between drug use and intoxicated sex is context-specific.

Our study also found that participants who had used drugs for three or more years were more likely to report sex under the influence of drugs than those who had used drugs for one year or less. Similar findings were found in men but not in women. The association was likely mediated by drug use behaviors among men since methamphetamine use among men had a stronger association with sex under the influence than that among women. The drug use and sexual risk behavior nexus was also confirmed in a previous study in Cambodia (Coupland et al., 2019).

Last, we found that the participants with drug rehabilitation experience were more likely to have sex under the influence of drugs. A similar result was found in men but not in women. In this study, 79.7% of men and 50% of women who had been to a drug rehabilitation center had been using drugs for at least three years, suggesting that long drug-use experience might have mediated the association among male PWUD. This finding points to the need for sexual risk education among male PWUD who had been to a drug rehabilitation center.

This study has some limitations. First, causality cannot be drawn as this study was cross-sectional and did not account for temporality. Second, we might have either over or underestimated the prevalence of sex under the influence of drugs because we used a single self-reported question that may lead to social desirability bias. Participants, particularly women, may shy away from reporting the experiences. Third, our study was not an event analysis, which limited us from drawing temporal relationships between covariates and outcome of interest. Last, our results were likely driven by selection bias. Only areas with a heavy burden of HIV and PWUD population were targeted for the data collection, and a token was provided to participants as compensation for their time.

### Conclusions

This study was the first to document a high prevalence of sex under the influence of drugs among PWUD in Cambodia. Several risk factors of sex under the influence of drugs were identified. The results point to the need for integrating HIV and harm-reduction programs using innovative approaches to address the overlapping risks in this key population. The programs may be devised with motivational and skills training (Calsyn, Cousins, et al., 2010; Calsyn, Crits-Christoph, et al., 2010) and conditional cash transfer to reduce drug use (Page et al., 2019). They should be specifically designed to address each identified risk factor to ameliorate the prevalence of sex under the influence of drugs, reducing the HIV incidence. The interventions should start with the population’s risk profiling to identify PWUD engaging in commercial sex, using methamphetamines, having used drugs for a more extended period, having been to a drug rehabilitation center, having a more extensive PWUD network, and having high psychological distress.

## Supplementary Information

Below is the link to the electronic supplementary material.

Supplementary file1 (DOCX 48 KB)

## Data Availability

The data used for this study are owned by the National Center for HIV/AIDS, Dermatology and STD. They cannot be made available in the manuscript, the additional files, or a public repository. However, they can be accessed upon request from the Principal Investigator, Dr. Siyan Yi (siyan@doctor.com).
