# Supplementary material for: Sex Under the Influence of Drugs Among People Who Use Drugs in Cambodia: Findings From a National Survey
Source: Arch Sex Behav. 2022 Feb 22;51(3):1461–70. doi: 10.1007/s10508-021-02243-x (PMC8917026; doi:10.1007/s10508-021-02243-x)
Supplement: Supplementary file 1 — Supplementary file1 (DOCX 48 KB) [file 10508_2021_2243_MOESM1_ESM.docx]

**Table S1** Socio-demographic characteristics of men who use drugs with and without sex under the influence of drugs in the past 3 months

| Socio-demographics |  | Sex under the influence of drugs | | |
| --- | --- | --- | --- | --- |
|  | Total  *(n*=675) | Yes  (*n*=267) | No  (*n*=408) |  |
|  | *n* (%) | *n* (%) | *n* (%) | P-value*^*^* |
| Living in urban area | 578 (85.6) | 250 (93.6) | 328 (80.4) | <0.001 |
| Age groups |  |  |  | 0.05 |
| 18–24 | 246 (36.5) | 82 (30.8) | 164 (40.2) |  |
| 25–34 | 282 (41.8) | 121 (45.5) | 161 (39.5) |  |
| 35+ | 146 (21.7) | 63 (23.7) | 83 (20.3) |  |
| Khmer ethnicity | 645 (95.8) | 254 (95.5) | 391 (96.1) | 0.71 |
| Marital status |  |  |  | 0.005 |
| Married | 226 (33.8) | 84 (31.6) | 142 (35.2) |  |
| Divorced/separated/widowed | 65 (9.7) | 38 (14.3) | 27 (6.7) |  |
| Never married | 378 (56.5) | 144 (54.1) | 234 (58.1) |  |
| Level of education |  |  |  | 0.40 |
| Primary (grades 0–6) | 299 (44.3) | 121 (45.3) | 178 (43.6) |  |
| Lower secondary (grades 7–9) | 193 (28.6) | 81 (30.3) | 112 (27.4) |  |
| Upper secondary or higher | 183 (27.1) | 65 (24.3) | 118 (28.9) |  |
| Living arrangement |  |  |  | 0.03 |
| With family | 365 (54.2) | 136 (51.1) | 229 (56.3) |  |
| Homeless | 75 (11.9) | 42 (15.8) | 33 (8.1) |  |
| Own home | 134 (19.9) | 54 (20.3) | 80 (19.7) |  |
| With friends | 49 (7.3) | 18 (6.8) | 31 (7.6) |  |
| Others | 50 (7.4) | 16 (6.0) | 34 (8.3) |  |
| Employment |  |  |  | 0.01 |
| Unemployed | 65 (9.6) | 35 (13.1) | 30 (7.3) |  |
| Entertainment worker | 23 (3.4) | 5 (1.9) | 18 (4.4) |  |
| Office/laborer/farmer | 355 (52.6) | 147 (55.1) | 208 (51.0) |  |
| Others | 232 (34.4) | 80 (29.9) | 152 (37.3) |  |
| Level of monthly income in USD | | | | 0.24 |
| <100 | 218 (32.3) | 96 (36.0) | 122 (29.9) |  |
| 100–199 | 326 (48.3) | 120 (44.9) | 206 (50.5) |  |
| 200+ | 131 (19.4) | 51 (19.1) | 80 (19.6) |  |

*^*^Chi-square (or Fisher’s exact test when a cell count was smaller than 5) was used.*

**Table S2** Socio-demographic characteristics of women who use drugs with and without sex under the influence of drugs in the past 3 months

| Socio-demographics |  | Sex under the influence of drugs | | |
| --- | --- | --- | --- | --- |
|  | Total  *(n*=472) | Yes  (*n*=189) | No  (*n*=283) |  |
|  | *n* (%) | *n* (%) | *n* (%) | P-value*^*^* |
| Living in urban area | 430 (91.1) | 186 (98.4) | 244 (86.2) | <0.001 |
| Age groups |  |  |  | 0.65 |
| 18–24 | 136 (28.8) | 50 (26.5) | 86 (30.4) |  |
| 25–34 | 231 (48.9) | 95 (50.2) | 136 (48.1) |  |
| 35+ | 105 (22.3) | 44 (23.3) | 61 (21.5) |  |
| Khmer ethnicity | 442 (94.4) | 174 (93.5) | 268 (95.0) | 0.49 |
| Marital status |  |  |  | 0.69 |
| Married | 229 (48.8) | 90 (48.1) | 139 (49.3) |  |
| Divorced/separated/widowed | 119 (25.4) | 45 (24.1) | 74 (26.2) |  |
| Never married | 121 (25.8) | 52 (27.8) | 69 (24.5) |  |
| Level of education |  |  |  | 0.65 |
| Primary (grades 0–6) | 327 (69.3) | 135 (71.4) | 192 (67.8) |  |
| Lower secondary (grades 7–9) | 118 (25.0) | 43 (22.7) | 75 (26.5) |  |
| Upper secondary or higher | 27 (5.7) | 11 (5.8) | 16 (5.6) |  |
| Living arrangement |  |  |  | 0.25 |
| With family | 126 (26.7) | 57 (30.2) | 69 (24.5) |  |
| Homeless | 37 (7.9) | 16 (8.5) | 21 (7.4) |  |
| Own home | 139 (29.5) | 45 (23.8) | 94 (33.3) |  |
| With friends | 87 (18.5) | 38 (20.1) | 49 (17.4) |  |
| Others | 82 (17.4) | 33 (17.4) | 49 (17.4) |  |
| Employment |  |  |  | 0.03 |
| Unemployed | 47 (9.9) | 20 (10.6) | 27 (9.5) |  |
| Entertainment worker | 188 (39.8) | 71 (37.6) | 117 (41.4) |  |
| Office/laborer/farmer | 73 (15.5) | 20 (10.6) | 53 (18.7) |  |
| Others | 164 (34.7) | 78 (41.2) | 86 (30.4) |  |
| Level of monthly income in USD | | | | 0.57 |
| <100 | 163 (34.5) | 60 (31.7) | 103 (36.4) |  |
| 100–199 | 224 (47.5) | 93 (49.2) | 131 (46.3) |  |
| 200+ | 85 (18.0) | 36 (19.1) | 49 (17.3) |  |

*^*^Chi-square (or Fisher’s exact test when a cell count was smaller than 5) was used.*

**Table S3** Sexual behaviors, substance use, and psychological distress among men who use drugs with and without sex under the influence of drugs in the past 3 months

|  |  | Sex under the influence of drugs | | |
| --- | --- | --- | --- | --- |
|  | Total  *(n*=675) | Yes (*n*=267) | No  (*n*=408) |  |
|  | *n* (%) | *n* (%) | *n* (%) | P-value |
| Number of sex partners in the past 3 months | | | | <0.001 |
| One partner | 334 (49.9) | 91 (34.3) | 243 (60.1) | <0.001 |
| 2–3 partners | 221 (33.0) | 104 (39.3) | 117 (28.9) | 0.006 |
| 4+ partners | 114 (17.1) | 70 (26.4) | 44 (11.0) | <0.001 |
| Age at first sex <18 | 233 (33.3) | 102 (38.5) | 121 (30.0) | 0.02 |
| Always used condoms in the past 3 months | 144 (21.4) | 57 (21.4) | 87 (21.4) | 0.99 |
| Having sex in exchange for money or gift in the past 3 months | 159 (23.6) | 85 (31.8) | 74 (18.1) | <0.001 |
| Type of drug used in the past three months | | | | |
| Methamphetamine | 512 (75.8) | 237 (88.8) | 275 (67.4) | <0.001 |
| Heroin | 75 (11.1) | 41 (15.4) | 34 (8.3) | 0.005 |
| Duration of drug use |  |  |  | <0.001 |
| Less than one year | 202 (29.9) | 54 (20.2) | 148 (36.3) | <0.001 |
| 1–2 years | 157 (23.3) | 56 (21.0) | 101 (24.7) | 0.26 |
| 3 years or more | 316 (46.8) | 157 (58.8) | 159 (39.0) | <0.001 |
| Been sent to drug rehabilitation in a life time | 118 (17.5) | 74 (27.7) | 44 (10.8) | <0.001 |
| Number drug users you know in the past 12 months | | | | <0.001 |
| 0–4 people | 144 (21.3) | 32 (12.0) | 112 (27.4) | <0.001 |
| 5–9 people | 160 (23.7) | 51 (19.1) | 109 (26.7) | 0.02 |
| 10+ people | 371 (55.0) | 184 (68.9) | 187 (45.8) | <0.001 |
| High level of psychological distress^†^ | 274 (40.6) | 133 (49.8) | 141 (34.6) | <0.001 |

*Abbreviations: GHQ, General Health Questionnaire; PWUD, people who use drugs.*

*^*^Chi-square test (or Fisher’s exact test when a cell count was smaller than 5) was used.*

^†^*Participants with the sum score of GHQ-12 above its mean were considered to have high psychological distress.*

**Table S4** Sexual behaviors, substance use, and psychological distress among women who use drugs with and without sex under the influence of drugs in the past 3 months

|  |  | Sex under the influence of drugs | | |
| --- | --- | --- | --- | --- |
|  | Total  *(n*=472) | Yes (*n*=189) | No  (*n*=283) |  |
|  | *n* (%) | *n* (%) | *n* (%) | P-value |
| Number of sex partners in the past 3 months | | | | <0.001 |
| One partner | 234 (50.3) | 46 (24.6) | 188 (67.6) | <0.001 |
| 2–3 partners | 94 (20.2) | 42 (22.5) | 52 (18.7) | 0.32 |
| 4+ partners | 137 (29.5) | 99 (52.9) | 38 (13.7) | <0.001 |
| Age at first sex <18 | 194 (41.3) | 92 (48.7) | 102 (36.3) | 0.01 |
| Always used condoms in the past 3 months | 141 (29.9) | 57 (30.2) | 84 (29.7) | 0.91 |
| Having sex in exchange for money or gift in the past 3 months | 252 (53.4) | 140 (74.1) | 112 (39.6) | <0.001 |
| Type of drug used in the past three months | | | | |
| Methamphetamine | 338 (71.6) | 160 (84.7) | 178 (62.9) | <0.001 |
| Heroin | 38 (8.0) | 13 (6.9) | 25 (8.8) | 0.44 |
| Duration of drug use |  |  |  | <0.001 |
| Less than one year | 180 (38.2) | 63 (33.5) | 117 (41.3) | 0.09 |
| 1–2 years | 157 (33.3) | 56 (29.8) | 101 (35.7) | 0.18 |
| 3 years or more | 134 (28.5) | 69 (36.7) | 65 (23.0) | 0.001 |
| Been sent to drug rehabilitation in a life time | 65 (13.8) | 38 (20.1) | 27 (9.5) | 0.001 |
| Number drug users you know in the past 12 months | | | | <0.001 |
| 0–4 people | 171 (36.2) | 49 (25.9) | 122 (43.1) | <0.001 |
| 5–9 people | 158 (33.5) | 55 (29.1) | 103 (36.4) | 0.10 |
| 10+ people | 143 (30.3) | 85 (45.0) | 58 (20.5) | <0.001 |
| High level of psychological distress^†^ | 235 (49.8) | 119 (63.0) | 116 (41.0) | <0.001 |

*Abbreviations: GHQ, General Health Questionnaire; PWUD, people who use drugs.*

*^*^Chi-square test (or Fisher’s exact test when a cell count was smaller than 5) was used.*

^†^*Participants with the sum score of GHQ-12 above its mean were considered to have high psychological distress.*

**Table S5** Risk factors associated with sex under the influence of drugs in the past 3 months among men who use drugs in bivariate and multiple logistic regression analyses

| Variables in the model | OR (95% CI) | P-value | AOR (95% CI) | P-value*^*^* |
| --- | --- | --- | --- | --- |
| Living area |  |  |  |  |
| Rural | Reference |  | Reference |  |
| Urban | 3.58 (2.07–6.21) | <0.001 | 2.56 (1.35–4.87) | 0.004 |
| Marital status |  |  |  |  |
| Married | Reference |  | Reference |  |
| Divorced/separated | 2.38 (1.35-4.17) | 0.003 | 1.54 (0.78-3.02) | 0.20 |
| Never married | 1.04 (0.74-4.17) | 0.82 | 0.91 (0.57-1.45) | 0.69 |
| Living arrangement |  |  |  |  |
| With family | Reference |  | Reference |  |
| Homeless | 2.14 (1.29-3.54) | 0.003 | 1.52 (0.80-2.90) | 0.20 |
| Own home | 1.13 (0.75-1.70) | 0.53 | 1.43 (0.83-2.46) | 0.20 |
| With friends | 0.98 (0.52-1.81) | 0.94 | 1.01 (0.49-2.08) | 0.97 |
| Others | 0.79 (0.42-1.49) | 0.47 | 0.74 (0.35-1.55) | 0.42 |
| Employment |  |  |  |  |
| Unemployed | Reference |  | Reference |  |
| Entertainment worker | 0.24 (0.08-0.72) | 0.01 | 0.33 (0.08-1.39) | 0.13 |
| Office/laborer/farmer | 0.60 (0.35-1.03) | 0.06 | 0.82 (0.40-1.66) | 0.58 |
| Others | 0.45 (0.26-0.79) | 0.005 | 0.52 (0.25-1.08) | 0.08 |
| Level of monthly income in USD | |  |  |  |
| <100 | Reference |  | Reference |  |
| 100–199 | 0.74 (0.52-1.05) | 0.09 | 0.81 (0.51-1.28) | 0.36 |
| 200+ | 0.81 (0.52-1.26) | 0.35 | 0.72 (0.41-1.28) | 0.26 |
| Number of sex partners in past three months | |  |  |  |
| One partner | Reference |  | Reference |  |
| 2–3 partners | 2.37 (1.66–3.39) | <0.001 | 2.28 (1.49–3.48) | <0.001 |
| 4+ partners | 4.25 (2.71–6.64) | <0.001 | 5.29 (3.00–9.31) | <0.001 |
| Age at first sex |  |  |  |  |
| Below 18 years old | Reference |  | Reference |  |
| 18 years and above | 0.68 (0.49–0.95) | 0.02 | 0.87 (0.58–1.31) | 0.52 |
| Having sex in exchange for money or gift in past 3 months | | | |  |
| No | Reference |  | Reference |  |
| Yes | 2.11 (1.47–3.02) | <0.001 | 1.87 (1.20–2.92) | 0.006 |
| Methamphetamine use in the past three months | |  |  |  |
| No | Reference |  | Reference |  |
| Yes | 3.82 (2.48–5.88) | <0.001 | 3.85 (2.28–6.51) | <0.001 |
| Heroin use in the past three months | |  |  |  |
| No | Reference |  | Reference |  |
| Yes | 1.99 (1.23-3.23) | 0.005 | 1.50 (0.78-2.90) | 0.22 |
| Duration of drug use |  |  |  |  |
| Less than one year | Reference |  | Reference |  |
| 1–2 years | 1.52 (0.97–2.39) | 0.07 | 1.97 (1.15–3.36) | 0.01 |
| 3 years or more | 2.71 (1.84–3.96) | <0.001 | 1.76 (1.07–2.90) | 0.02 |
| Been sent to drug rehabilitation in a lifetime | | |  |  |
| No | Reference |  | Reference |  |
| Yes | 3.17 (2.10–4.79) | <0.001 | 1.97 (1.16–3.36) | 0.01 |
| Number drug users you know in the past 12 months | | |  |  |
| 0–4 people | Reference |  | Reference |  |
| 5–9 people | 1.64 (0.98–2.74) | 0.06 | 1.45 (0.79–2.65) | 0.22 |
| 10+ people | 3.44 (2.21–5.36) | <0.001 | 1.71 (1.00–2.90) | 0.05 |
| Level of psychological distress^†^ | | | |  |
| Low | Reference |  | Reference |  |
| High | 1.88 (1.37–2.57) | <0.001 | 1.38 (0.95–2.01) | 0.09 |

*Abbreviations: CI, confidence interval; AOR, adjusted odds ratio; OR, odds ratio.*

*^*^Variables associated with sex under the influence of drugs in the bivariate analyses at a level of p<0.05 were simultaneously included in the model.*

^†^*Participants with the sum score of the General Health Questionnaire (GHQ-12) above its mean were considered to have high psychological distress.*

**Table S6** Risk factors associated with sex under the influence of drugs in the past 3 months among women who use drugs in bivariate and multiple logistic regression analyses

| Variables in the model | OR (95% CI) | P-value | AOR (95% CI) | P-value*^*^* |
| --- | --- | --- | --- | --- |
| Living area |  |  |  |  |
| Rural | Reference |  | Reference |  |
| Urban | 9.91 (3.01–32.56) | <0.001 | 7.87 (1.28–48.21) | 0.03 |
| Living arrangement |  |  |  |  |
| With family | Reference |  | Reference |  |
| Homeless | 0.92 (0.44-1.93) | 0.83 | 0.83 (0.30-2.04) | 0.62 |
| Own home | 0.58 (0.35-0.95) | 0.03 | 0.81 (0.42-1.55) | 0.53 |
| With friends | 0.94 (0.54-1.63) | 0.82 | 1.18 (0.57-2.44) | 0.65 |
| Others | 0.81 (0.46-1.43) | 0.48 | 0.52 (0.26-1.07) | 0.08 |
| Employment |  |  |  |  |
| Unemployed | Reference |  | Reference |  |
| Entertainment worker | 0.82 (0.43-1.56) | 0.55 | 0.27 (0.12-0.63) | 0.002 |
| Office/laborer/farmer | 0.51 (0.23-1.10) | 0.09 | 0.52 (0.20-1.34) | 0.18 |
| Others | 1.22 (0.63-2.35) | 0.54 | 0.48 (0.22-1.07) | 0.07 |
| Number of sex partners in past three months | |  |  |  |
| One partner | Reference |  | Reference |  |
| 2–3 partners | 3.30 (1.96–5.54) | <0.001 | 3.09 (1.53–6.23) | 0.002 |
| 4+ partners | 10.65 (6.50–17.44) | <0.001 | 10.96 (4.96–24.21) | <0.001 |
| Age at first sex |  |  |  |  |
| Below 18 years old | Reference |  | Reference |  |
| 18 years and above | 0.60 (0.41–0.87) | 0.01 | 0.87 (0.54–1.41) | 0.58 |
| Having sex in exchange for money or gift in past 3 months | | | |  |
| No | Reference |  | Reference |  |
| Yes | 4.36 (2.91–6.53) | <0.001 | 1.13 (0.56–2.29) | 0.72 |
| Methamphetamine use in the past three months | |  |  |  |
| No | Reference |  | Reference |  |
| Yes | 3.25 (2.05–5.17) | <0.001 | 2.37 (1.33–4.23) | 0.003 |
| Duration of drug use |  |  |  |  |
| Less than one year | Reference |  | Reference |  |
| 1–2 years | 1.03 (0.66–1.61) | 0.90 | 0.93 (0.52–1.65) | 0.78 |
| 3 years or more | 1.97 (1.25–3.11) | 0.004 | 1.36 (0.83–2.79) | 0.17 |
| Been sent to drug rehabilitation in a lifetime | | |  |  |
| No | Reference |  | Reference |  |
| Yes | 2.38 (1.40–4.06) | 0.001 | 1.19 (0.61–2.33) | 0.60 |
| Number drug users you know in the past 12 months | | |  |  |
| 0–4 people | Reference |  | Reference |  |
| 5–9 people | 1.33 (0.83–2.12) | 0.23 | 1.04 (0.58–1.88) | 0.88 |
| 10+ people | 3.65 (2.28–5.84) | <0.001 | 2.35 (1.29–4.27) | 0.005 |
| Level of psychological distress^†^ | | | |  |
| Low | Reference |  | Reference |  |
| High | 2.45 (1.67–3.57) | <0.001 | 1.98 (1.21–3.25) | <0.001 |

*Abbreviations: CI, confidence interval; AOR, adjusted odds ratio; OR, odds ratio.*

*^*^Variables associated with sex under the influence of drugs in the bivariate analyses at a level of p<0.05 were simultaneously included in the model.*

^†^*Participants with the sum score of the General Health Questionnaire (GHQ-12) above its mean were considered to have high psychological distress.*
